# Effect of statin treatment on mortality in elderly patients with type 2 diabetes mellitus patients: a retrospective cohort study

**DOI:** 10.1186/s12877-023-04252-y

**Published:** 2023-09-11

**Authors:** Yao Fan, Juan Wang, Haidi Wu, Lingli Dai, Lan Wang, Liubao Gu

**Affiliations:** 1 Division of Clinical Epidemiology, Geriatric Hospital of Nanjing Medical University, Nanjing, China; 2 Clinical Trial Center, Geriatric Hospital of Nanjing Medical University, Nanjing, China; 3 Department of Endocrinology and Metabolism, Geriatric Hospital of Nanjing Medical University, Nanjing, China; 4 Department of Cardiovascular Medicine, Geriatric Hospital of Nanjing Medical University, Nanjing, China

**Keywords:** Type 2 diabetes mellitus, Statins, Cohort study, Mortality, Elderly

## Abstract

**Background:**

The effects of statins on the reduction of mortality in individuals aged 75 years or older remain controversial. We conducted this study to investigate whether there is an association between statin therapy and mortality in patients with type 2 diabetes mellitus (T2DM) who are over the age of 75 years.

**Methods:**

The present study used data from the Staged Diabetes Targeting Management Study, which began in 2005. A total of 518 T2DM patients older than 75 years were included. Cox regression analyses were used to evaluate the association between statins and specific causes of death in patients with T2DM.

**Results:**

After a follow-up period of 6.09 years (interquartile range 3.94–8.81 years), 111 out of 518 patients died. The results of Cox regression analyses showed that there was no significant association between statin use and all-cause mortality (HR 0.75; 95% CI 0.47, 1.19) after adjustment for all potential confounders. Subgroup analysis indicated that statins had no association with the risk of all-cause mortality or deaths caused by ischemic cardiovascular diseases in T2DM patients with or without coronary heart disease.

**Conclusions:**

Our study found no significant association between all-cause mortality and statin use in T2DM patients over the age of 75 years. More evidence is needed to support the use of statins in the elderly T2DM patients.

**Supplementary Information:**

The online version contains supplementary material available at 10.1186/s12877-023-04252-y.

## Background

Atherosclerotic cardiovascular diseases (ASCVD) are the leading cause of death in most parts of the world [1, 2], with coronary heart disease (CHD) and ischemic stroke (IS) accounting for 42% and 35% of global cardiovascular mortality, respectively [2]. Statins are first-line evidence-based drugs for the management of dyslipidemia and for secondary prevention of ASCVD events across age groups [3, 4]. A number of studies have demonstrated that in addition to its cholesterol lowering effect, statins also show pleiotropic effects such as modulating immune responses, and inhibiting subclinical inflammation and oxidative stresses [5, 6]. In addition, the benefits of statins for primary prevention in subjects under 75 years old have been well established based on multiple randomized clinical trials (RCTs), except for those over 75 years of age [7–10].

The advantage of statins for primary prevention of cardiovascular events and mortality in patients over 75 years old remains controversial, mainly because there is significantly less evidence for this age group and the risk for statin-related harms increases with age, which could potentially offset their positive effects [11–15]. Most of the available evidence regarding statin use for primary prevention of ASCVD in these patients is derived from subgroup analyses of RCTs. However, the US Preventive Services Task Force concluded in a recent review that older people are underrepresented in trials and there is insufficient evidence to draw a robust conclusion about the balance between benefits and harms of statins for primary prevention in this age group [11, 12]. Recently, it was revealed by an individual participant data meta-analysis including 28 RCTs that statin therapy resulted in a significant reduction of major vascular events irrespective of age, but there is less evidence of benefit among participants older than 75 years without previous vascular diseases (primary prevention) [15]. Consistently, two recently published real-world retrospective studies also found that use of statins was not associated with a lower risk of outcomes including all-cause death in the primary prevention among individuals without diabetes or other modifiable risk factors [13, 14].

It was well known that patients with type 2 diabetes mellitus (T2DM) have a similar risk of ASCVD to those with a history of cardiovascular disease [16]. Interestingly, statin use was significantly associated with reduced incident ASCVD and all cause mortality in diabetic patients without clinically recognized ASCVD in Spanish population [13]. Against this background, we undertook this study to investigate whether there is an association between statin therapy and mortality in patients with T2DM over the age of 75 years in Chinese population.

## Methods

### Patients

The present study was conducted using the data taken from the Staged Diabetes Targeting Management (SDTM) Study [17, 18], which was started since 2005 as a continuous structured diabetes care program in Jiangsu Province Official Hospital. All patients were managed according to the Staged Diabetes Management protocol adopted from International Diabetes Center (Minneapolis, US) [19], and the information of each visit was recorded online (www.chinasdtm.com). The study protocol conforms to the ethical guidelines of the 1975 Declaration of Helsinki and has been approved by the Ethical Committee, Jiangsu Province Official Hospital, Nanjing. Informed consent was obtained from all patients at the time of first assessment to allow use of their data for research purposes.

### Clinical and laboratory data

The information recorded in the SDTM study has been described [20, 21]. Briefly, body weight, height and blood pressure were measured by the diabetic specialist nurses according to standard protocols. Body mass index (BMI) was calculated as the ratio of the weight (kg) to squared height (m^2^). Details on personal information, history of disease and current use of medications were also obtained from all patients through interviews by the nurses. Blood tests were carried out after an overnight fasting for glucose, lipid profiles, uric acid (UA), renal/liver functions and glycated hemoglobin (HbA1c). Glucose, total cholesterol (TC), triglycerides (TG), high-density lipoprotein cholesterol (HDL-C), low-density lipoprotein cholesterol (LDL-C), and serum creatinine (Scr) were measured using Hitach 7060 automated analyzer (Hitachi Koki Co. Ltd., Hitachinaka City, Japan). HbA1c was measured by Bio-rad Diamat high-performance liquid chromatography analyzer (Bio-Rad Labs., Brea, CA, USA). Estimated glomerular filtration rate (eGFR) was calculated using the CKD-EPI equation [22].

### Outcomes

The incident endpoint events of all-cause mortality and the causes of death including ischemic cardiovascular diseases (CHD and IS), cardiovascular and cerebrovascular disease (CCVD) (including IS, CHD and hemorrhagic stroke (HS)), cancer, respiratory system disease and renal failure were collected from the SDTM database. In addition, telephone-interviews were performed by diabetic specialist nurses to confirm the status of 518 participants in January 2018. Finally, all the mortality data were further verified through the resident database from local centers for disease control system as well.

### Statistical analysis

Statistical analysis was conducted using the SPSS (version 20.0) software. Variables were assessed for the full cohort and stratified by statin use. All data are expressed as mean ± standard deviation, median (interquartile range) or percentage, where appropriate. Unpaired student’s t-test was used to compare differences between two groups. Rates were compared using the *χ* ^*2*^ test. The effect of statins on all-cause mortality, and deaths caused by ischemic cardiovascular disease, HS, cancer, respiratory system disease and renal failure were analyzed and in subgroups stratified by the presence or absence of prior CHD. The hazard ratio (HR) and 95% confidence interval (CI) by Cox regression were used to estimate the association between statins and specific death of T2DM. Variables those were significant in univariable analyses and had biological plausibility were entered into multivariate Cox regression models.

## Results

### Characteristics of study population

**Fig. 1:**
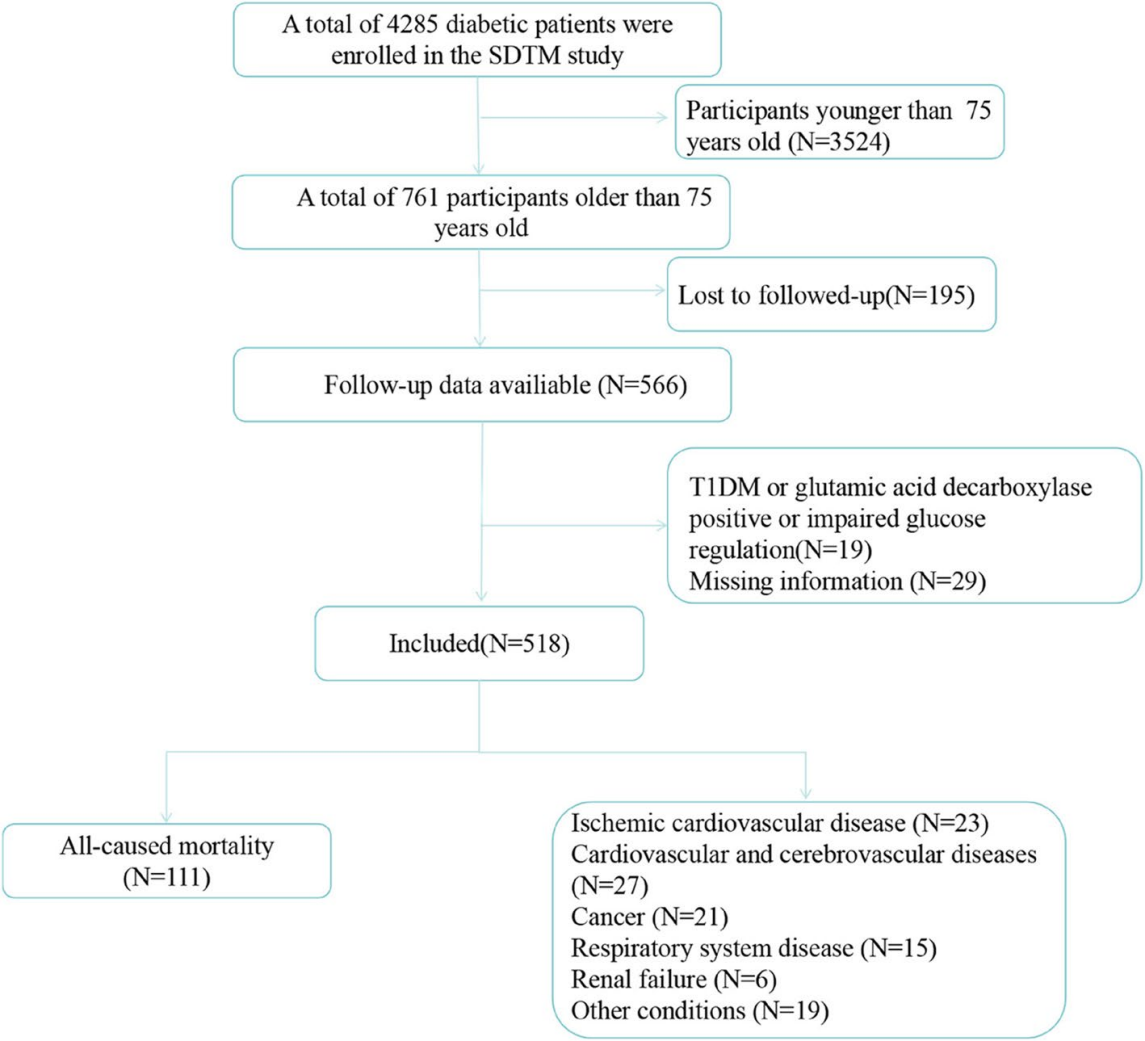
Flow chart of participants included and excluded in the analyses

A total of 4285 diabetic patients were enrolled in the SDTM study until January 2018. After excluding those who younger than 75 years at baseline (*N* = 3524), loss to follow-up (*N* = 195), had a history of type 1 diabetes, glutamic acid decarboxylase positive or impaired glucose regulation at baseline (*N* = 19), missing data for key variables (*N* = 29), we had the complete data of 518 patients with T2DM for the final analysis (Fig. 1). Characteristics of participants included in and excluded from the study were shown in Table S1.

Of the 518 participants, there were 307 (59.27%) men and 211 (40.73%) women, with a mean age of 79.82 ± 3.50 years. The clinical and metabolic characteristics of the subjects grouped by the use of statins were shown in Table 1. Generally, the subjects using statins have higher levels of BMI, systolic blood pressure (SBP), fasting blood glucose (FBG), postprandial blood glucose (PBG), HbA1c levels, and lower hemoglobin (Hb) levels (Table 1).

**Table 1:** Baseline characteristics of the study population according to using of statins

	**Before PSM**			***p*** **-value**	**After PSM**			***p*** **-value**
**Characteristics**	**All subjects**	**Statins**	**Without Stains**		**All subjects**	**Statins**	**Without Stains**	
	***n*** ** = 518**	***N*** ** = 228**	***N*** ** = 290**		***n*** ** = 232**	***N*** ** = 116**	***N*** ** = 116**	
Sex (male, %) ^a^	307(59.27)	118 (51.75)	189 (65.17)	0.002^*^	141 (60.78)	69 (59.48)	72 (62.07)	0.687
Age (years) ^b^	79.82 ± 3.50	80.24 ± 3.76	79.48 ± 3.25	0.016^*^	80.59 ± 3.79	80.48 ± 4.00	80.70 ± 3.57	0.650
BMI (kg/m^2^) ^b^	24.58 ± 3.52	25.13 ± 3.73	24.15 ± 3.29	0.002^*^	24.58 ± 3.40	24.46 ± 3.31	24.70 ± 3.50	0.597
Smoking (yes, %) ^a^	60 (11.58)	24 (10.53)	36 (12.41)	0.517	29 (12.50)	12 (10.34)	17 (14.66)	0.321
Drinking (yes, %) ^a^	38 (7.34)	16 (7.02)	22 (7.59)	0.805	12 (5.17)	4 (3.45)	8 (6.90)	0.236
SBP (mmHg) ^b^	133.22 ± 15.54	134.92 ± 16.37	131.87 ± 14.74	0.027^*^	134.08 ± 16.55	134.09 ± 17.17	134.06 ± 15.97	0.987
DBP (mmHg) ^b^	73.61 ± 9.39	74.25 ± 9.79	73.11 ± 9.04	0.172	73.14 ± 9.74	73.74 ± 9.75	72.54 ± 9.75	0.350
Microalbuminuria (mg/g) ^c^	26.00 (12.00, 68.00)	29.70 (14.60, 74.30)	21.00 (10.35, 65.00)	0.094	22.41 (11.03, 68.00)	26.66 (13.98, 63.01)	18.00 (10.00, 75.50)	0.309
Hb(g/L) ^b^	127.14 ± 16.54	125.32 ± 14.95	128.95 ± 17.84	0.022^*^	127.7 ± 15.56	127.54 ± 14.41	127.86 ± 16.70	0.876
FBG (mmol/L) ^b^	7.33 ± 2.32	7.63 ± 2.48	7.09 ± 2.16	0.010^*^	7.27 ± 2.35	7.46 ± 2.57	7.09 ± 2.12	0.233
PBG (mmol/L) ^b^	12.47 ± 4.43	13.44 ± 4.57	11.61 ± 4.12	< 0.001^*^	12.64 ± 4.16	12.71 ± 4.25	12.58 ± 4.09	0.823
HbA1c (mmol/L) ^b^	7.72 ± 1.86	8.24 ± 2.04	7.30 ± 1.59	< 0.001^*^	7.81 ± 1.80	7.83 ± 1.75	7.79 ± 1.85	0.873
TC (mmol/L) ^b^	4.50 ± 1.09	4.48 ± 1.19	4.52 ± 1.00	0.702	4.33 ± 1.09	4.37 ± 1.20	4.30 ± 0.98	0.603
TG (mmol/L) ^b^	1.50 ± 0.89	1.45 ± 0.79	1.54 ± 0.97	0.292	1.48 ± 0.91	1.46 ± 0.90	1.50 ± 0.92	0.717
HDL-C (mmol/L) ^b^	1.15 ± 0.35	1.14 ± 0.31	1.16 ± 0.39	0.642	1.10 ± 0.35	1.12 ± 0.25	1.08 ± 0.42	0.360
LDL-C (mmol/L) ^b^	2.66 ± 0.88	2.64 ± 0.99	2.67 ± 0.78	0.652	2.54 ± 0.89	2.54 ± 1.02	2.53 ± 0.73	0.925
eGFR [mL/(min·1.73 m^2^)] ^b^	69.04 ± 16.59	69.65 ± 16.86	68.51 ± 16.38	0.448	69.32 ± 15.72	70.87 ± 16.62	67.78 ± 14.69	0.137
AST (U/L) ^b^	21.68 ± 9.29	20.82 ± 7.98	22.44 ± 10.26	0.054	20.53 ± 7.92	20.81 ± 8.50	20.22 ± 7.30	0.581
ALT (U/L) ^b^	20.56 ± 17.81	19.46 ± 12.30	21.49 ± 21.38	0.208	19.12 ± 11.52	19.39 ± 12.74	18.85 ± 10.18	0.721
GTT (U/L) ^c^	22.00 (15.00, 33.00)	23.00 (16.00, 35.00)	21.00 (14.00, 32.00)	0.190	21.5 (15.00, 33.25)	22.00 (15.00, 35.00)	21.00 (14.00, 32.00)	0.574
UA (mmol/L)^b^	341.96 ± 93.70	338.21 ± 91.62	345.18 ± 95.51	0.411	337.59 ± 88.90	331.69 ± 87.68	343.49 ± 90.10	0.313
Medical history								
CHD (yes, %) ^a^	127 (24.52)	70 (30.70)	57 (19.66)	0.004^*^	54 (23.28)	27 (23.28)	27 (23.28)	> 0.999
HT (yes, %) ^a^	349 (67.37)	178 (78.07)	171 (58.97)	< 0.001^*^	162 (69.83)	80 (68.97)	82 (70.69)	0.775
Medication								
Calcium channel blocker (yes, %) ^a^	194 (37.45)	90 (39.47)	104 (35.86)	0.399	97 (41.81)	48 (41.38)	49 (42.24)	0.894
Angiotensin Receptor Blocker (yes, %) ^a^	225 (43.44)	116 (50.88)	109 (37.59)	0.002^*^	115 (49.57)	54 (46.55)	61 (52.59)	0.358
Sulfonylureas (yes, %) ^a^	152(29.34)	53 (23.25)	99 (34.14)	0.007^*^	58 (25.00)	29 (25.00)	29 (25.00)	> 0.999
Insulin (yes, %) ^a^	167 (32.24)	89 (39.04)	78 (26.90)	0.003^*^	90 (38.79)	46 (39.66)	44 (37.93)	0.788
Metformin (yes, %) ^a^	121 (23.36)	71 (31.14)	50 (17.24)	< 0.001^*^	52 (22.41)	28 (24.14)	24 (20.69)	0.529
Beta blockers (yes, %) ^a^	122 (23.55)	69 (30.26)	53 (18.28)	0.001^*^	55 (23.71)	27 (23.28)	28 (24.14)	0.877
Aspirin(yes, %) ^a^	294 (56.76)	176 (77.19)	118 (40.69)	< 0.001^*^	156 (67.24)	77 (66.38)	79 (68.10)	0.780

*Abbreviations*: *PSM* propensity score match, *BMI* body mass index, *SBP* systolic blood pressure, *DBP* diastolic blood pressure, *Hb* hemoglobin, *FBG* fasting blood glucose, *PBG* postprandial blood glucose, *HbA1c* glycated hemoglobin, *TC* total cholesterol, *TG* triglycerides, *HDL-C* high-density lipoprotein cholesterol, *LDL-C* low-density lipoprotein cholesterol, *eGFR* estimated glomerular filtration rate, *AST* aspartate transaminase, *ALT* alanine transaminase, *GTT* glutamyltranspeptidase, *UA* uric acid, *CHD* coronary heart disease, *HT* hypertension

^*^ *p*-value < 0.05

^a^Data are expressed as Number (percentage), *p*-values refer to Chi square test

^b^Data are expressed as Mean (SD), *p*-values refer to t test

^c^Non-parameter Mann–Whitney U-Test., *p*-value was shown in the table

Furthermore, we performed propensity score matching (PSM) model by key variables at baseline, including sex, age, BMI, SBP, Hb, HbA1c, HDL-C, CHD, HT, calcium channel blocker, angiotensin receptor blocker, sulfonylureas, insulin, metformin, beta blockers. After PSM, 116 pairs of participants were included. There were no difference of variables between groups (Table 1).

### Characteristics of population with different outcomes

The median follow-up period was 6.09 years (interquartile range 3.94–8.81 years). During the follow-up, 111 patients died, including 23 caused by ischemic cardiovascular disease, 27 by CCVD, 21 by cancer, 15 by respiratory system disease, 6 by renal failure and 19 by other conditions. As shown in Table 2, age, microalbuminuria, PBG, and UA showed higher levels in the group of patients who died, while HDL-C and eGFR showed lower levels.

**Table 2:** Baseline characteristics of the study population according to death

Characteristics	Survival	Death	*p-*value
	***N*** ** = 407**	***N*** ** = 111**	
Sex (male, %) ^a^	234 (57.49)	73 (65.77)	0.116
Age (years) ^b^	79.62 ± 3.38	80.54 ± 3.86	0.023^*^
BMI (kg/m^2^) ^b^	24.55 ± 3.51	24.68 ± 3.58	0.746
Smoking (yes, %) ^a^	48 (11.79)	12 (10.81)	0.768
Drinking (yes, %) ^a^	34 (8.35)	4 (3.60)	0.089
SBP (mmHg) ^b^	132.81 ± 15.56	134.73 ± 15.47	0.252
DBP (mmHg) ^b^	73.50 ± 9.54	74.01 ± 8.83	0.616
Microalbuminuria (mg/g) ^c^	21.97 (10.75, 48.87)	53.00 (18.00, 161.00)	< 0.001^*^
Hb(g/L) ^b^	127.73 ± 15.59	125.16 ± 19.35	0.173
FBG (mmol/L) ^b^	7.27 ± 2.16	7.54 ± 2.82	0.290
PBG (mmol/L) ^b^	12.12 ± 4.23	13.84 ± 4.94	0.004^*^
HbA1c (mmol/L) ^b^	7.65 ± 1.85	7.98 ± 1.88	0.101
TC (mmol/L) ^b^	4.54 ± 1.09	4.37 ± 1.07	0.173
TG (mmol/L) ^b^	1.50 ± 0.88	1.50 ± 0.91	0.959
HDL-C (mmol/L) ^b^	1.17 ± 0.36	1.09 ± 0.34	0.029^*^
LDL-C (mmol/L) ^b^	2.67 ± 0.90	2.62 ± 0.81	0.648
eGFR [mL/(min·1.73 m^2^)] ^b^	70.25 ± 15.39	64.66 ± 19.80	0.008^*^
AST (U/L) ^b^	21.71 ± 9.48	21.58 ± 8.60	0.894
ALT (U/L) ^b^	20.73 ± 18.98	19.93 ± 12.86	0.677
GTT (U/L) ^c^	22.00 (15.00, 32.25)	23.00 (15.00, 36.00)	0.436
UA (mmol/L)^b^	336.29 ± 88.82	362.13 ± 107.38	0.023^*^
Medical history			
CHD (yes, %) ^a^	95 (23.34)	32 (28.83)	0.234
HT (yes, %) ^a^	266 (65.36)	83 (74.77)	0.061
Medication			
Calcium channel blocker (yes, %) ^a^	150 (36.86)	44 (39.64)	0.591
Angiotensin Receptor Blocker (yes, %) ^a^	171 (42.01)	54 (48.65)	0.211
Sulfonylureas (yes, %) ^a^	127 (31.20)	25 (22.52)	0.075
Insulin (yes, %) ^a^	115 (28.26)	52 (46.85)	< 0.001^*^
Metformin (yes, %) ^a^	97 (23.83)	24 (21.62)	0.625
Statins (yes, %) ^a^	188 (46.19)	40 (36.04)	0.056
Beta blockers (yes, %) ^a^	94 (23.10)	28 (25.23)	0.639
Aspirin (yes, %) ^a^	229 (56.27)	65 (58.56)	0.666

*Abbreviations*: *BMI* body mass index, *SBP* systolic blood pressure, *DBP* diastolic blood pressure, *Hb* hemoglobin, *FBG* fasting blood glucose, *PBG* postprandial blood glucose, *HbA1c* glycated hemoglobin, *TC* total cholesterol, *TG* triglycerides, *HDL-C* high-density lipoprotein cholesterol, *LDL-C* low-density lipoprotein cholesterol, *eGFR* estimated glomerular filtration rate, *AST* aspartate transaminase, *ALT* alanine transaminase, *GTT* glutamyltranspeptidase, *UA* uric acid, *CHD* coronary heart disease, *HT* hypertension

^*^ *p*-value < 0.05

^a^Data are expressed as Number (percentage), *p*-values refer to Chi square test

^b^Data are expressed as Mean (SD), *p*-values refer to t test

^c^Non-parameter Mann–Whitney U-Test., *p*-value was shown in the table

### Association of statin use and all-cause mortality and specific mortality

There was no statistical association between statin use and all-cause mortality (HR 0.75; 95% CI 0.47, 1.19) and CCVD (HR 0.49; 95% CI 0.18, 1.32) after adjustment for all potential confounders including baseline age, sex, BMI, Hb, HbA1c, medical history and medications. Statin use was associated with reduced ischemic cardiovascular disease mortality (HR 0.31; 95% CI 0.10, 0.97). However, after PSM, Cox regression analyses showed that statin use was not associated with all-caused mortality, ischemic cardiovascular disease mortality and CCVD mortality (Table 3).

**Table 3:** Association analysis of statins and all-cause mortality and specific causes of death

Outcomes	Before PSM				After PSM	
	***N***	**HR** ^**a**^ ** (95% CI)**	**HR** ^**b**^ ** (95% CI)**	**HR** ^**c**^ ** (95% CI)**	***N***	**HR (95% CI)**
Overall	111	1.10 (0.74, 1.63)	1.01 (0.68, 1.51)	0.75 (0.47, 1.19)	61	0.96 (0.56, 1.64)
Ischemic Cardiovascular Disease (IS + CHD)	23	0.61 (0.24,1.56)	0.56 (0.21, 1.45)	0.31 (0.10, 0.97)^*^	12	0.70 (0.21, 2.39)
CCVD (IS + CHD + HS)	27	0.86 (0.38, 1.94)	0.79 (0.34, 1.79)	0.49 (0.18, 1.32)	14	1.03 (0.35, 3.06)
Cancer	21	1.17 (0.48, 2.88)	1.20 (0.48, 2.97)	1.25 (0.44, 3.55)	12	1.10 (0.33, 3.65)
Respiratory System Disease	15	0.81 (0.25, 2.65)	0.72 (0.21, 2.39)	0.52 (0.14, 1.98)	8	1.05 (0.24, 4.59)
Renal Failure	6	2.14 (0.42, 10.90)	2.41 (0.47, 12.26)	2.86 (0.03, 319.30)	2	1.60 (0.09, 27.53)

*Abbreviations*: *PSM* propensity score match, *IS* ischemic stroke, *CHD* coronary heart disease, *CCVD* cardiovascular and cerebrovascular disease, *HS* hemorrhagic stroke, *BMI* body mass index, *SBP* systolic blood pressure, *Hb* hemoglobin, *HbA1c* glycated hemoglobin, *HT* hypertension

^*^ *p*-value < 0.05

^a^HR value for crude

^b^Adjusted for age and sex

^c^Adjusted for age, sex, BMI, Hb, HbA1c, CHD, HT, angiotensin receptor blocker, sulfonylureas, insulin, metformin, beta blockers, aspirin

Subgroup analysis indicated that statins had no association with the all-caused mortality or mortality caused by ischemic cardiovascular disease in T2DM patients without CHD, HRs (95% CIs) were 0.59 (0.33, 1.03) and 0.31 (0.09, 1.06), respectively.In T2DM patients with CHD, statins had no association with all-caused mortality [1.79 (0.62, 5.20)], neither (Table 4). Similar results were also shown by cox regression analyses in matched pairs.

**Table 4:** Stratification association analysis of statins and death by CHD disease history

Subgroup	Outcomes	Before PSM				After PSM	
		***N***	**HR** ^**a**^ ** (95% CI)**	**HR** ^**b**^ ** (95% CI)**	**HR** ^**c**^ ** (95% CI)**	***N***	**HR (95% CI)**
Without CHD	Overall	79	0.93 (0.57, 1.53)	0.86 (0.52, 1.42)	0.59 (0.33, 1.03)	43	0.66 (0.34, 1.29)
	Ischemic Cardiovascular Disease (IS)	17	0.58 (0.19, 1.79)	0.52 (0.17, 1.63)	0.31 (0.09, 1.06)	10	0.55 (0.14, 2.19)
	CCVD (IS + HS)	19	0.68 (0.24, 1.91)	0.60 (0.21, 1.70)	0.41 (0.13, 1.26)	11	0.73 (0.21, 2.54)
CHD	Overall	32	1.48 (0.73, 3.03)	1.53 (0.72, 3.25)	1.79 (0.62, 5.20)	18	2.44 (0.89, 6.63)
	Ischemic Cardiovascular Disease (IS + CHD)	6	0.75 (0.13, 4.28)	0.80 (0.14, 4.69)	-	2	2.64 (0.13, 53.45)
	CCVD (IS + CHD + HS)	8	1.34 (0.32, 5.60)	1.53 (0.36, 6.55)	1.73 (0.07, 41.04)	3	4.60 (0.36, 59.70)

Abbreviations: *PSM* propensity score match, *IS* ischemic stroke, *CHD* coronary heart disease, *CCVD* cardiovascular and cerebrovascular disease, *HS* hemorrhagic stroke, *BMI* body mass index, *SBP* systolic blood pressure, *Hb* hemoglobin, *HbA1c* glycated hemoglobin, *HT* hypertension

^a^HR value for crude

^b^Adjusted for age and sex

^c^Adjusted for age, sex, BMI, Hb, HbA1c, HT, angiotensin receptor blocker, sulfonylureas, insulin, metformin, beta blockers, aspirin

## Discussions

Our findings explored issues that have remained controversial and insufficiently studied to date. In our cohort study of 518 T2DM patients over 75 years old, the results indicated a nonsignificant association of reduced all-caused mortality and ischemic cardiovascular disease with the statin use. To our knowledge, this is the first time to raise the possibility of statins associated with a reduction in mortality in elderly individuals in the Chinese population.

The potential benefits of statins for primary prevention of mortality in the elderly remain controversial. A subanalysis of the JUPITER study found no benefits of statins in reducing mortality for individuals aged > 70 years [23]. Similarly, in the PROSPER study that focused on primary prevention in elderly individuals, pravastatin was found to have no benefits for all-cause mortality [24]. Meta-analyses also suggested that statins do not have a protective effect against all-cause mortality for individuals aged over 65 years old in the setting of primary prevention [25, 26]. However, a nonsignificant direction toward increased all-cause mortality with pravastatin was observed among adults 75 years and older in the ALLHAT-LLT study [27].

In contrast, statins are associated with reduced mortality in aged 75 and older population in some other studies. In the US veterans study with patients 75 years and older and free of ASCVD at baseline, prescription of statins for the first time was significantly associated with a lower risk of all-cause and cardiovascular mortality [28]. The SCOPE-75 study suggested a remarkable reduction in the relative risk of all-cause death among statin users aged over 75 years for primary prevention [29]. The Reykjavik Study, which enrolled subjects with a mean age of 77 years, reported a greater benefit of statins in the subgroup of diabetic subjects [30]. Moreover, in a large-scale retrospective cohort study with 46, 864 people aged 75 years or more without cardiovascular diseases, Rafel Ramos and colleagues also revealed that statin use was significantly associated with reduced all-cause mortality in diabetic patients for primary prevention [13]. In fact, Rafel et al. found that the protective effect of statins against all-cause mortality in participants with diabetes became weaker as age increased and began to lose statistical significance at age 82 years [13].

Our findings appear to be consistent with these studies in elderly T2DM patients. In our population, statin use showed a reduction association with ischemic cardiovascular disease mortality, although it does not reach statistical significance after PSM. Age should be considered as an important factor affecting the protective effects of statins. It is worth noting that the average age in our study is about 80 years. Meanwhile, relatively small sample size may also responsible for the lack of effect observed.

The majority of published data suggest that statin usage does not affect the incidence of most cancers [31]. Our present study, in accordance with previous studies, demonstrates that the associations between statin use and cancer-related outcomes were not statistically significant. Similarly, non-vascular death, deaths caused by respiratory system disease and renal failure were not affected by statin use in the present study or in most previous studies.

There are several limitations in this study. It was carried out in a cohort that only comprised Chinese T2DM patients managed at a single outpatient clinic in Nanjing with a relative small sample size. There may be potential bias between different groups due to the observational study design, although we conducted PSM. More studies, especially prospective studies with large sample size and RCTs, are needed to confirm our finding. In addition, the proportion of patients who used low- or high-intensity statins was too small to investigate the possibility of different clinical outcomes between these groups. Despite these limitations, these real-world data recorded most of potential confounding factors, which might strengthen the findings of the present study. Moreover, the relatively long follow-up period (about 6.09 years) may provide a more accurate view of the effects of statin use on long-term mortality.

In conclusion, our findings suggest that statin use showed a possible reduction in all-cause mortality and ischemic cardiovascular disease mortality, although it does not reach statistical significance. More evidence is needed to support the use of statins in the elderly T2DM patients.

### Supplementary Information

**Additional file 1: Table S1. **Characteristics of participants included in and excluded from study.

## Data Availability

Data can be obtained from the corresponding author upon reasonable request.

## Abbreviations

ASCVD: Atherosclerotic cardiovascular diseases
CHD: Coronary heart disease
IS: Ischemic stroke
RCTs: Randomized clinical trials
T2DM: Type 2 diabetes mellitus
SDTM: Staged Diabetes Targeting Management
BMI: Body mass index
UA: Uric acid
HbA1c: Glycated hemoglobin
TC: Total cholesterol
TG: Triglycerides
HDL-C: High-density lipoprotein cholesterol
LDL-C: Low-density lipoprotein cholesterol
Scr: Serum creatinine
eGFR: Estimated glomerular filtration rate
CCVD: Cardiovascular and cerebrovascular disease
HS: Hemorrhagic stroke
HR: Hazard ratio
CI: Confidence interval
FBG: Fasting blood glucose
PBG: Postprandial blood glucose
Hb: Hemoglobin
PSM: Propensity score match
