# Supplementary material for: Effect of statin treatment on mortality in elderly patients with type 2 diabetes mellitus patients: a retrospective cohort study
Source: BMC Geriatr. 2023 Sep 11;23:549. doi: 10.1186/s12877-023-04252-y (PMC10494359; doi:10.1186/s12877-023-04252-y)
Supplement: Supplementary file 1 — Additional file 1: Table S1. Characteristics of participants included in and excluded from study. [file 12877_2023_4252_MOESM1_ESM.docx]

**Supplementary information**

After excluding those who younger than 75 years at baseline (N=3524), there are 761 participants. We further compared characteristics of participants included in study (*N*=518) with participants excluded from study (*N*=243). There were no significant differences in all characteristics between the two groups, except systolic blood pressure, microalbuminuria and alanine transaminase.

**Table S1.** Characteristics of participants included in and excluded from study.

| Characteristics | Total | Included in study | Excluded from study | p-value |
| --- | --- | --- | --- | --- |
|  | **n**=761 | **N**=518 | **N**=243 |  |
| Sex (male, %) ^a^ | 446(58.61) | 307(59.27) | 139(57.20) | 0.590 |
| Age (years) ^b^ | 79.83±3.56 | 79.77±3.49 | 79.95±3.71 | 0.517 |
| BMI (kg/m2) ^b^ | 24.65±3.47 | 24.58±3.52 | 24.81±3.36 | 0.390 |
| Smoking (yes, %) ^a^ | 86(11.30) | 61(11.78) | 25(10.29) | 0.546 |
| Drinking (yes, %) ^a^ | 53(6.96) | 38(7.34) | 15(6.17) | 0.557 |
| SBP (mmHg) ^b^ | 132.25±16.51 | 133.22±15.54 | 130.19±18.28 | 0.018* |
| DBP (mmHg) ^b^ | 73.41±9.37 | 73.61±9.39 | 72.97±9.33 | 0.382 |
| Microalbuminuria (mg/g) ^c^ | 23.00(11.00, 56.00) | 26.00(12.00, 68.00) | 18.65(10.00, 42.16) | 0.009* |
| Hb(g/L) ^b^ | 126.46±16.50 | 127.14±16.54 | 124.90±16.32 | 0.120 |
| FBG (mmol/L) ^b^ | 7.34±2.28 | 7.33±2.32 | 7.35±2.20 | 0.928 |
| PBG (mmol/L) ^b^ | 12.51±4.57 | 12.47±4.43 | 12.61±4.86 | 0.719 |
| HbA1c (mmol/L) ^b^ | 7.76±1.92 | 7.72±1.86 | 7.85±2.03 | 0.397 |
| TC (mmol/L) ^b^ | 4.49±1.09 | 4.50±1.09 | 4.45±1.09 | 0.590 |
| TG (mmol/L) ^b^ | 1.49±0.90 | 1.50±0.89 | 1.47±0.92 | 0.729 |
| HDL-C (mmol/L) ^b^ | 1.16±0.35 | 1.15±0.35 | 1.18±0.34 | 0.359 |
| LDL-C (mmol/L) ^b^ | 2.64±0.88 | 2.66±0.88 | 2.60±0.88 | 0.435 |
| eGFR [mL/(min·1.73 m2)] ^c^ | 69.22±16.72 | 69.04±16.59 | 69.60±17.00 | 0.671 |
| AST (U/L) ^b^ | 21.46±8.80 | 21.68±9.29 | 21.00±7.69 | 0.340 |
| ALT (U/L) ^b^ | 19.86±15.46 | 20.56±17.81 | 18.37±8.37 | 0.025* |
| GTT (U/L) ^c^ | 21.00(15.00, 32.00) | 22.00(15.00, 33.00) | 21.00(15.00, 30.00) | 0.161 |
| UA (mmol/L)^b^ | 340.38±96.88 | 341.96±93.70 | 336.96±103.56 | 0.520 |
| Medical history |  |  |  |  |
| CHD (yes, %) ^a^ | 182(23.92) | 127(24.52) | 55(22.63) | 0.570 |
| HT (yes, %) ^a^ | 506(66.49) | 349(67.37) | 157(64.61) | 0.451 |
| Medication |  |  |  |  |
| Calcium channel blocker (yes, %) ^a^ | 291(38.24) | 194(37.45) | 97(39.92) | 0.514 |
| Angiotensin Receptor Blocker (yes, %) ^a^ | 329(43.23) | 225(43.44) | 104(42.80) | 0.868 |
| Sulfonylureas (yes, %) ^a^ | 223(29.30) | 152(29.34) | 71(29.22) | 0.972 |
| Insulin (yes, %) ^a^ | 245(32.19) | 167(32.24) | 78(32.10) | 0.969 |
| Metformin (yes, %) ^a^ | 177(23.26) | 121(23.36) | 56(23.05) | 0.924 |
| Statins (yes, %) ^a^ | 340(44.68) | 228(44.02) | 112(46.09) | 0.591 |
| Beta blockers (yes, %) ^a^ | 173(22.73) | 122(23.55) | 51(20.99) | 0.431 |
| Aspirin(yes, %) ^a^ | 424(55.72) | 294(56.76) | 130(53.50) | 0.399 |
|  |  |  |  |  |
| Abbreviations: BMI body mass index, SBP systolic blood pressure, DBP diastolic blood pressure, Hb hemoglobin, WBC white blood cell, FBG fasting blood glucose, PBG postprandial blood glucose, HbA1c glycated hemoglobin, TC total cholesterol, TG triglycerides, HDL-C high-density lipoprotein cholesterol, LDL-C low-density lipoprotein cholesterol, eGFR estimated glomerular filtration rate, AST aspartate transaminase, ALT alanine transaminase, GTT glutamyltranspeptidase, UA uric acid, CHD coronary heart disease, HT hypertension. | | | | |
| ^a^ Data are expressed as Number (percentage), p-values refer to Chi square test; | | | | |
| ^b^ Data are expressed as Mean (SD), p-values refer to t test; | | | | |
| ^c^ Non-parameter Mann-Whitney U-Test., p-value was shown in the table. | | | | |
| ^*^p-value <0.05 | | | | |
